# Prognostic value of uPAR expression and angiogenesis in primary and metastatic melanoma

**DOI:** 10.1371/journal.pone.0210399

**Published:** 2019-01-14

**Authors:** Emilia Hugdahl, Ingeborg M. Bachmann, Cornelia Schuster, Rita G. Ladstein, Lars A. Akslen

**Affiliations:** 1 Centre for Cancer Biomarkers CCBIO, Department of Clinical Medicine, University of Bergen, Bergen, Norway; 2 Department of Dermatology, Haukeland University Hospital, Bergen, Norway; 3 Centre for Cancer Biomarkers CCBIO, Department of Clinical Science, University of Bergen, Bergen, Norway; 4 Department of Oncology Haukeland University Hospital, Bergen, Norway; 5 Department of Pathology, Haukeland University Hospital, Bergen, Norway; University of Pécs Medical School, HUNGARY

## Abstract

Angiogenesis is important for the progression of cutaneous melanoma. Here, we analyzed the prognostic impact of the angiogenic factor urokinase plasminogen activator resecptor (uPAR), vascular proliferation index (VPI) and tumor necrosis as a measure of hypoxia in a patient series of nodular melanomas (n = 255) and matched loco-regional metastases (n = 78). Expression of uPAR was determined by immunohistochemistry and VPI was assessed by dual immunohistochemistry using Factor-VIII/Ki67 staining. Necrosis was recorded based on HE-slides. As novel findings, high uPAR expression and high VPI were associated with each other, and with increased tumor thickness, presence of tumor necrosis, tumor ulceration, increased mitotic count and reduced cancer specific survival in primary melanoma. In matched cases, VPI was decreased in metastases, whereas the frequency of necrosis was increased. Our findings demonstrate for the first time the impact on melanoma specific survival of uPAR expression and VPI in primary tumors, and of increased necrosis as an indicator of tumor hypoxia in loco-regional metastases. These findings support the importance of tumor angiogenesis in melanoma aggressiveness, and suggest uPAR as an indicator of vascular proliferation and a potential biomarker in melanoma.

## Introduction

Angiogenesis is the formation of new capillaries from existing blood vessels, and sustained angiogenesis is a pathological feature in tumors as proposed by Folkman in 1971 [1]. Today, angiogenesis is regarded as a hallmark of cancer [2], and microvessel density (MVD) was introduced as a method of morphological assessment of intra-tumor neovascularization in 1991 [3].

In melanoma, angiogenesis is involved in tumor progression and metastasis [4, 5], and MVD has been shown to be an adverse prognostic factor [6–8]. Recently, vascular proliferation index, VPI, has been introduced as a novel angiogenesis marker showing superior prognostic value compared to MVD in endometrial, prostate, breast and lung cancer [9–12]. The hypothesis is that this method captures the actively expanding vasculature better than standard microvessel density, and that tumors with a higher VPI are more aggressive with poorer prognosis. Previously, proliferating endothelial cells was suggested as a prognostic factor in a small series of primary melanoma [13], and in a cohort of melanoma sentinel lymph node metastases [14].

Urokinase plasminogen activator receptor (uPAR) has a proteolytic function as a degrader of extracellular matrix [15], and it has been associated with invasion and metastasis in melanoma [16–21]. In addition, uPAR support endothelial cell proliferation, migration and permeability [22–24]. Whereas uPAR has shown prognostic value in different types of cancer, the association with survival has not been analyzed in melanoma [25–30].

The prognosis for stage III melanoma is heterogenous, with 10-year survival rates varying from 24–88% [31]. Presence of necrosis, related to tumor hypoxia and a prognostic histopathologic characteristic previously demonstrated for primary melanoma by our group [32, 33], has not been investigated as a survival marker in metastatic melanoma.

Here, we studied for the first time the presence and prognostic significance of uPAR expression in cutaneous melanoma, and whether vascular proliferation by VPI was associated with uPAR, tumor necrosis, and melanoma specific survival. We also examined potential differences between primary tumors and matched loco-regional metastases.

## Material and methods

### Patients

This patient series consists of 255 consecutive cases of primary nodular cutaneous melanomas diagnosed at the Department of Pathology, Haukeland University Hospital (Bergen, Norway) during 1998–2008 [34, 35]. The presence of a vertical growth phase and absence of a radial growth phase, *i*.*e*. adjacent *in situ* or microinvasive components, were used as inclusion criteria. Cases with minor secondary involvement of the adjacent epidermis up to three epidermal ridges were included. There was no known history of familial occurrence. During this time period, the sentinel node procedure was not performed in Norway, and our series therefore lacks complete staging. Median age was 70 years, and the median primary tumor thickness was 3.6 mm (range 0.7–29.0 mm). Complete information on patient survival, time and cause of death was available in all 255 cases. Last date of follow-up of survival, with information on cause of death from the Cancer Registry of Norway, was December 31, 2014. Median follow-up time for survivors was 115 months (range 72–203 months). During the follow-up period, 88 patients (35%) died of malignant melanoma and 76 (30%) died of other causes. Last date of follow-up of recurrent disease, including information from medical records, was February 29, 2016. A summary of patient characteristics is given in S1 Table.

In addition, 78 paired biopsies from the first appearing local (skin; n = 26) or regional metastatic tumor (lymph nodes; n = 52) in this series were examined. In cases with multiple metastases at the same time within the same organ compartment, we have selected the largest tumor. If the largest metastasis contained necrosis to the extent that representative tissue could not be sampled, then the largest tumor without such necrosis was chosen. Of the 78 cases with metastases, HE-slides of tissue biopsies were available in 73 cases (n = 25 skin metastases, n = 48 lymph node metastases), while in five cases only HE-slides of core needle biopsies were available. Further, due to limited tumor tissue in the paraffin blocks, 69 of the 73 cases were available for whole slide immunohistochemistry, and 68 of the 73 cases were available for tissue microarrays.

The patient series used in this study lacks written consent, and this was approved by the Norwegian Data Inspectorate and the Regional Committee for Ethics in Research (Health Region III; 178.05) (REK 2009/564). All data were fully anonymized. The study was performed in accordance with the Declaration of Helsinki Principles.

### Clinico-pathologic variables

Clinical data and histologic variables of primary melanoma were included: date of histologic diagnosis, sex, age at diagnosis, tumor anatomic site, tumor thickness according to Breslow [36], level of invasion according to Clark [37], mitotic count, the mitotic marker PHH3 [38], microscopic tumor ulceration [38] and tumor necrosis [32].

For all loco-regional metastases, clinical data on date of histologic diagnosis and site of first metastasis were registered. All slides of the loco-regional metastases were examined (EH, LAA) and the following histologic variables were recorded: maximum tumor diameter and tumor necrosis [32]

### Tissue microarray (TMA)

The TMA technique has been described and validated in several studies [39–41]. Three tissue cylinders (diameter 1.0 mm) from representative tumor areas identified on H&E stained slides, such as the supra-basal area of the primary melanomas, were punched from archival blocks and mounted into a recipient paraffin block using a custom made precision instrument (for TMAs of primary melanoma: Beecher Instruments, Silver Spring, MD, USA, for TMAs of melanoma metastases: Minicore 3, Tissue Arrayer, Alphelys, France). Sections (5 μm) of the resulting TMA blocks were made by standard technique.

### uPAR immunohistochemistry

The immunohistochemical staining was performed on 5 μm TMA sections of paraffin-embedded archival tissue. Sufficient tumor tissue for immunohistochemistry was available in 248 of the primary melanoma cases and 68 of the loco-regional metastatic melanomas. The slides were dewaxed with xylene/ethanol. Antigen retrieval was performed for 20 minutes in Target Retrieval Solution (DAKO 1699) (pH = 6) in microwave. Endogenous peroxidase activity was prevented by treating the slides with peroxidase block (DAKO S2001) for 8 minutes. The slides were incubated with the mouse monoclonal antibody uPAR (dilution 1:100) (ADG 3937, Sekisui Diagnostics, Pfungstadt, Germany) overnight at 4°C. EnVision labelled polymer method was then used (DAKO K4001 or K4003) for 30 minutes. 3-amino-9-ethylcarbazole (AEC) (DAKO K3469) was used as substrate chromogen. Brief counterstaining was performed with hematoxylin (DAKO S2020). Negative control was obtained by omitting the primary antibody.

### Dual Factor-VIII/Ki67immunohistochemistry

The immunohistochemical staining was performed on 5 μm sections of paraffin-embedded archival tissue. Sufficient tumor tissue for immunohistochemistry was available in 242 of the primary melanomas and 69 of the paired loco-regional metastases. Staining was first performed in primary melanoma by IMB, and the staining protocol was adjusted (antibody dilution and incubation time) to obtain an optimal result when metastases were subsequently stained by EH. The slides were dewaxed with xylene/ethanol before microwave antigen retrieval for 20 minutes in Target Retrieval Solution (DAKO 1699) (pH = 6). Endogenous peroxidase activity was prevented by treating the slides with peroxidase block (DAKO S2001) for 8 minutes. The slides were simultaneously incubated with the two antibodies Factor-VIII (polyclonal rabbit antibody, DAKO A0082) (dilution 1:500 for primary melanoma and 1:1000 for metastases) and Ki67 (monoclonal mouse antibody, DAKO M7240) (dilution 1:200 for primary melanoma and 1:50 for metastases) for 60 minutes in primary melanoma and 90 minutes in metastases. The staining procedure was performed using the secondary goat anti-mouse antibody (1031–04, Southern Biotech, Birmingham, AL, USA) diluted in anti-rabbit EnVision labelled polymer method (DAKO K4003) at 1:100 for 30 minutes. Visualization was done with the Ferangie Blue TM Chromagen System (Biocare Medical, Concord, CA, USA) (applied for 20 minutes) for Ki67 and with 3-amino-9-ethylcarbazole (AEC) (DAKO K3469) as substrate chromogen for Factor-VIII (applied for 10 minutes). No counterstaining was applied. Negative controls were obtained by omitting the primary antibody.

### Evaluation of uPAR expression

Expression of uPAR was characterized by membrane-associated staining of melanoma cells. The staining intensity was recorded as either negative, weak, moderate or strong (0–3). Proportion of tumor cells stained was recorded as either 1 (< 10%), 2 (10–50%) or 3 (> 50%). A staining index (SI) was calculated as the product of staining intensity and area score (proportion of tumor cells stained) [42]. For additional evaluation of the staining, a subset of cases (n = 54) was scored blindly by two observers (EH, RGL) showing good inter-observer agreement (κ = 0.63, p < 0.01). Evaluation of the cases was done blinded for patient characteristics and outcome.

### Evaluation of microvessel density (MVD) and vascular proliferation index (VPI)

In tumor tissue sections, blood vessel endothelial cells were visualized using immunohistochemistry for both an endothelial marker (Factor-VIII) and a proliferation marker (Ki67). The tissue sections were examined at low magnification to identify the most vascular areas of the tumor (“hot-spots”). In primary melanoma, the hot-spot regions were almost exclusively localized around the invasive front at the tumor base. In melanoma metastases, the hot-spot area was marked by a pathologist (LAA). Within these hot-spots, a maximum of 10 fields at x 400 were examined and the number of vessels counted (MVD). Minimum one-half of the high power field (HPF) (x400) must contain viable tumor cells. Vessels more than one-half HPF (x400) below the invasive front, or vessels close (< 1 HPF) to ulcerated or necrotic areas were not counted. Any highlighted endothelial cell or cell cluster clearly separate from adjacent microvessels were regarded as a distinct countable microvessel. In addition, the number of proliferating vessels were recorded in the same 10 fields within the hot-spot by counting the number of vessels with endothelial cells co-expressing the endothelial and the proliferation markers (pMVD). VPI is defined as the ratio between the number of proliferating vessels and the total number of vessels. MVD and pMVD was assessed by IMB in primary melanoma, and by EH in the melanoma metastases.

To compensate for any inter-observer difference, 25 of the primary melanomas were assessed for MVD and pMVD also by EH. The vessel counts showed good inter-observer agreement (κ = 0.68, p < 0.01 for MVD, κ = 0.61, p < 0.01 for pMVD and κ = 0.84, p < 0.01 for VPI). The median values for MVD, pMVD and VPI among the 25 primary melanomas counted by EH were then compared to IMBs median values, and a ratio was calculated for MVD (1.17), pMVD (1.03) and VPI (0.91) (S2 Table). These ratios were then used as correction factors on IMBs counts for MVD, pMVD and VPI in primary melanoma.

### Statistics

Statistical analyses were performed using the IBM Statistical package for the Social Sciences version 23 (IBM Corp. Armonk, NY). Associations between different categorical variables were assessed by the Pearson’s chi-square test. Comparison of two or more continuous variables not following the normal distribution was performed using the Mann-Whitney U or Kruskal-Wallis tests. Differences between paired samples were determined by the McNemar test for categorical data and Wilcoxon test for continuous variables. Kappa (κ) statistics was used in analyses of inter-observer agreement of categorical data.

Univariate analyses of time to death due to malignant melanoma were performed using the product-limit procedure (Kaplan-Meier method), and differences between categories were estimated by the log-rank test, with date of histological diagnosis as the starting point for analyses of primary melanomas and date of first metastasis as the starting point for analyses of metastatic melanomas. Patients who died of other causes were censored at the date of death. The influence of co-variates on patient survival was analyzed by the proportional hazards method, and tested by the likelihood ratio (lratio) test. The variables were tested by log-log plot to determine their ability to be incorporated in multivariate models. All results were considered significant if p < 0.05.

In the statistical analyses, the cut-off points for categorization of staining index data were determined after considering the frequency distribution curve, size of possible subgroups and number of events. The cut-off points for categorization of continuous variables were determined after considering the survival curve patterns of the quartiles. As a rule, subgroups with similar survival were merged. When one quartile`s survival curve diverged from the three other survival curves, the cut-off was set at this quartile. In the case of an even distribution of survival curves, or a divergence between the survival curves of the two lowest quartiles versus the two highest quartiles, the median was used as cut-off point.

## Results

### Expression of uPAR in primary and metastatic tumors

**Fig 1:**
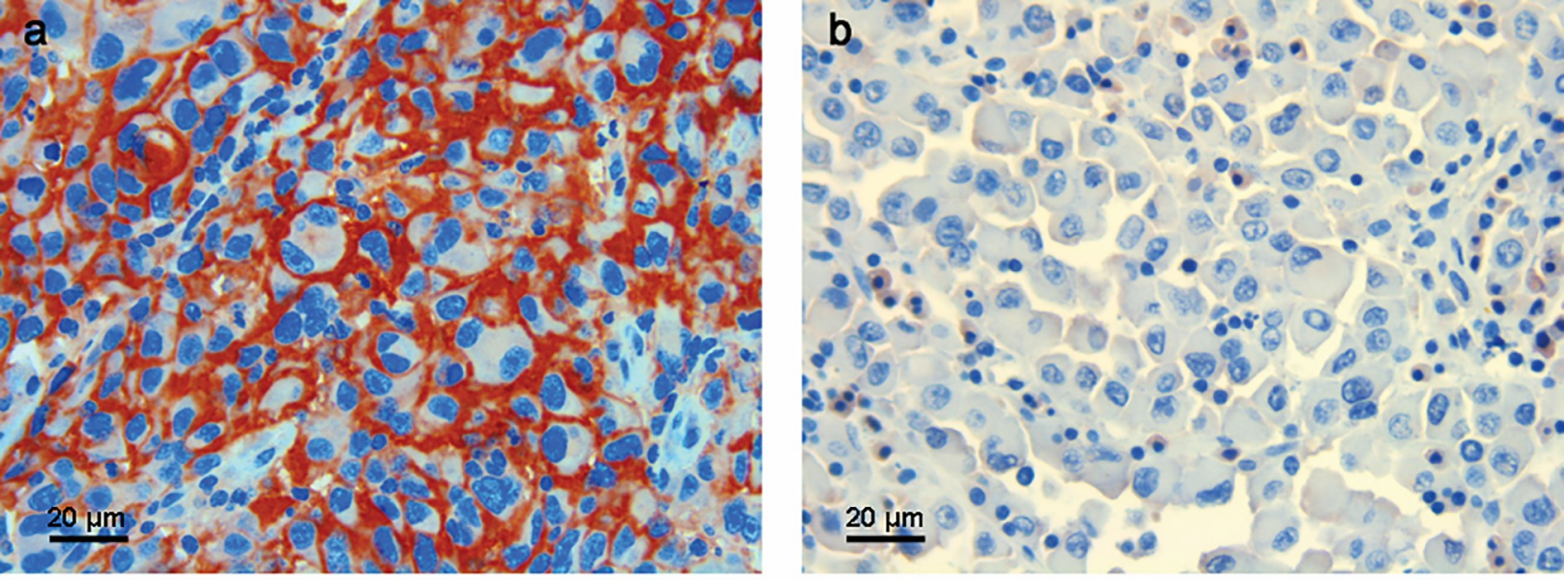
uPAR expression. Immunohistochemical staining of a uPAR positive primary melanoma (a) (x400) and a uPAR expression negative loco-regional metastasis (b) (x 400).

Immunohistochemical staining obtained by the uPAR antibody was assessed in primary melanomas (n = 248) and 68 paired loco-regional metastases. The staining was membrane-associated and generally homogenous, and differences in staining intensity between tumor areas (heterogeneity) were rarely observed. Protein expression was categorized according to the staining index (SI) as either negative/low or positive/high, with a cut-off value based on the median (uPAR; SI = 4) in primary melanomas (Fig 1).

Positive uPAR expression in primary melanoma was associated with increased VPI (p = 0.01), increased tumor thickness (p < 0.01), presence of tumor ulceration (p = 0.03), increased mitotic count (p < 0.01) and presence of tumor necrosis (p = 0.01) (Table 1).

**Table 1:** uPAR protein expression in association with vascular proliferation index (VPI) and histopathologic characteristics in primary melanoma (n = 248).

	uPAR expression (SI)^a^^.^^c^		
	low	high	p-value
**VPI**			0.01^b^
median	8.3	12.7	
**Tumor thickness (mm)**			< 0.01^b^
median	3.0	4.5	
**Tumor ulceration**^**d**^			0.03^e^
absent (n)	70	43	
Present (n)	62	67	
**Mitotoc count (per mm**^**2**^**)**			< 0.01^b^
median	2.8	8.0	
**Tumor necrosis**			0.01^e^
absent (n)	110	75	
present (n)	24	37	

Abbreviation: SI; staining index. ns; non significant

^**a**^Cut-off point: median

^b^Mann-Whitney U test

^**c**^2 missing cases

^d^4 missing cases

^e^Chi-square test

In loco-regional metastases, uPAR was significantly associated with increased MVD (p < 0.05; Mann -Whitney test), but not with VPI (S3 Table).

Regarding differences between primary tumors and the first appearing loco-regional metastasis (n = 65), uPAR expression was not significantly different in metastases compared to primary tumors, although it tended to be lower (p = 0.27; McNemar test) (S4 Table).

### Angiogenesis markers in primary and metastatic tumors

**Fig 2:**
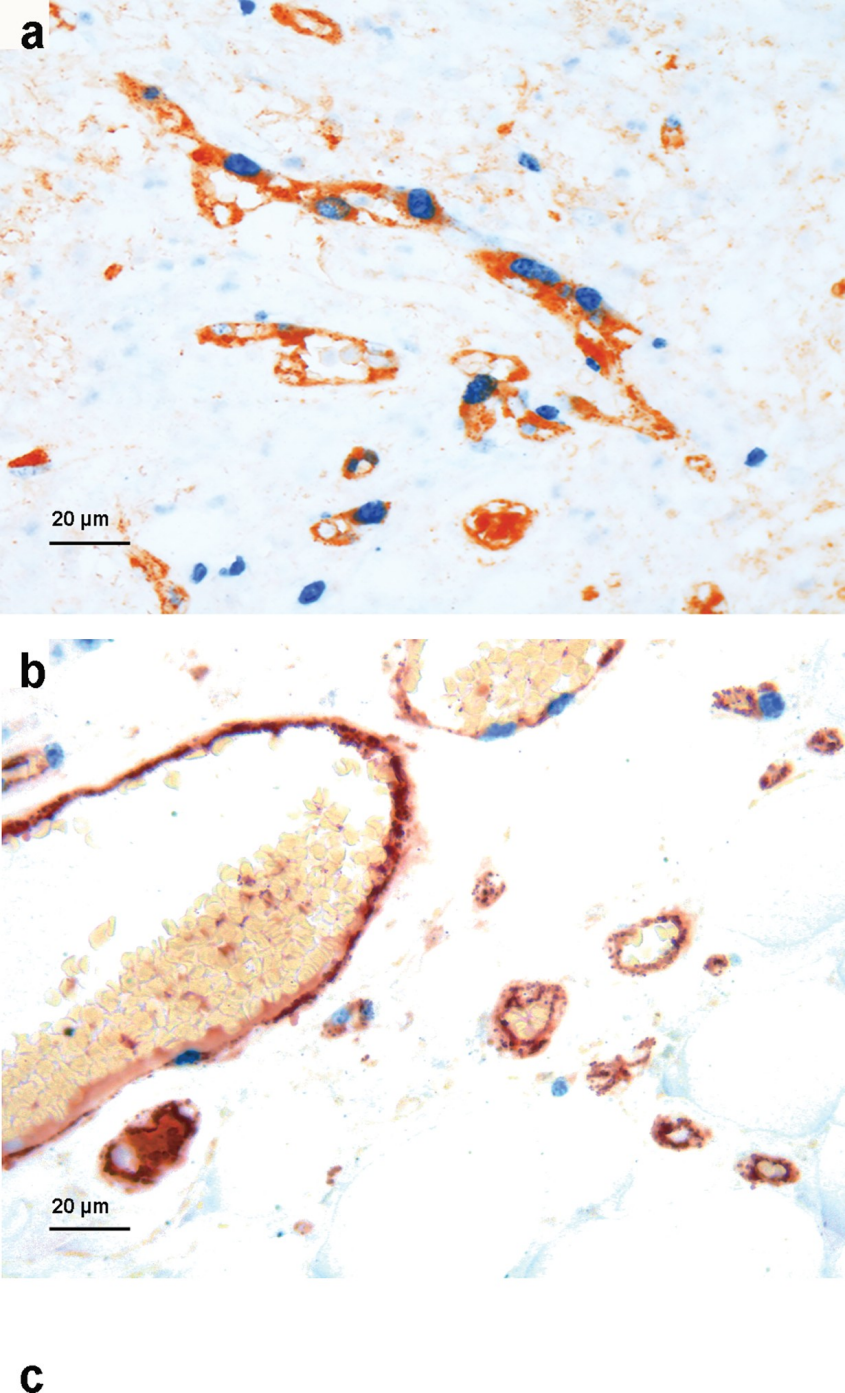
Vascular proliferation. IHC dual staining with Factor-VIII and Ki67 showing proliferating vessels in primary melanoma (a) (x400) and loco-regional metastasis (b) (x400).

In primary melanoma (n = 242), median MVD was 62/mm^2^, pMVD 6.1/mm^2^ and VPI 9.6%. In loco-regional metastases (n = 69), median MVD was 95/mm^2^, pMVD 9.0/mm^2^ and VPI 7.6% (Table 2). For information on MVD, pMVD and VPI specifically in skin or lymph node metastases, see Table 2. MVD, pMVD and VPI in primary and metastatic melanoma were categorized as either low or high based on the median (MVD and pMVD) or the lower quartile (VPI, 5.7%) in primary tumors (Fig 2). Median pMVD was significantly higher among primary melanoma with loco-regional metastasis (pMVD = 7.6) than among cases without metastasis (pMVD = 5.6) (p = 0.024, Mann Whitney U test), whereas there were no significant differences for MVD or VPI (S5 Table).

**Table 2:** Distribution of MVD, pMVD and VPI in primary (n = 242) and metastatic (n = 69) melanoma.

	Primary melanoma	Metastatic melanoma	Skin metastases (n = 24)	Lymph node metastases (n = 45)
**MVD** median (no/mm^2^)	62.3	95.0	91.3	98.7
**pMVD** median (no/mm^2^)	6.1	9.0	7.8	10.0
**VPI** median (%)	9.6	7.6	11.1	7.4

There was sufficient tumor tissue in both the primary and matched metastatic lesions in 67 cases. Among these, median MVD was significantly higher in metastases: 97/mm^2^ versus 67/mm^2^ in primary melanoma (p < 0.01). The opposite was found for VPI, which had significantly lower median value in metastases; 7.6% versus 11.0% in primary melanoma (p < 0.05), whereas there was no significant difference in median pMVD between primary melanomas and paired metastases (p = 0.36). A similar pattern was found among the 44 cases of paired primary and lymph node tumors, while among the 23 cases with paired primary and skin tumors there was no significant reduction of VPI in metastases (S6 Table).

Increased VPI in primary melanomas was significantly associated with features of aggressive tumors, like increased tumor thickness (p < 0.01), presence of tumor ulceration (p < 0.01), high mitotic count (p < 0.01) and presence of tumor necrosis (p < 0.01) (Table 3).

**Table 3:** VPI in association with histopathologic characteristics of primary melanoma (n = 242).

	VPI (median)	p-value
**Tumor thickness (mm)**		< 0.01^b^
< 2.0 mm	5.2	
2.0–4.1 mm	9.5	
> 4.0 mm	13.5	
**Tumor ulceration**		< 0.01^c^
absent	6.4	
present	13.2	
**Mitotic count (per mm**^**2**^**)**^**a**^		< 0.01^c^
low	7.5	
high	10.5	
**Tumor necrosis**		< 0.01^c^
absent	8.3	
present	14.5	

^a^Cut-off point: lower quartile

^b^ Kruskal Wallis test

^c^Mann -Whitney U test

### Tumor necrosis in loco-regional metastases

In this study, tumor necrosis was focused due to its known association with tumor hypoxia, and also because it is a prognostic factor in primary melanoma [32, 33]. Of the 78 matched and first appearing loco-regional metastases, 26 were from skin/subcutaneous tissue, and 52 were lymph-node metastases. All variables were recorded based on HE-slides of tissue biopsies (n = 73; 25 skin metastases and 48 lymph node metastases).

**Fig 3:**
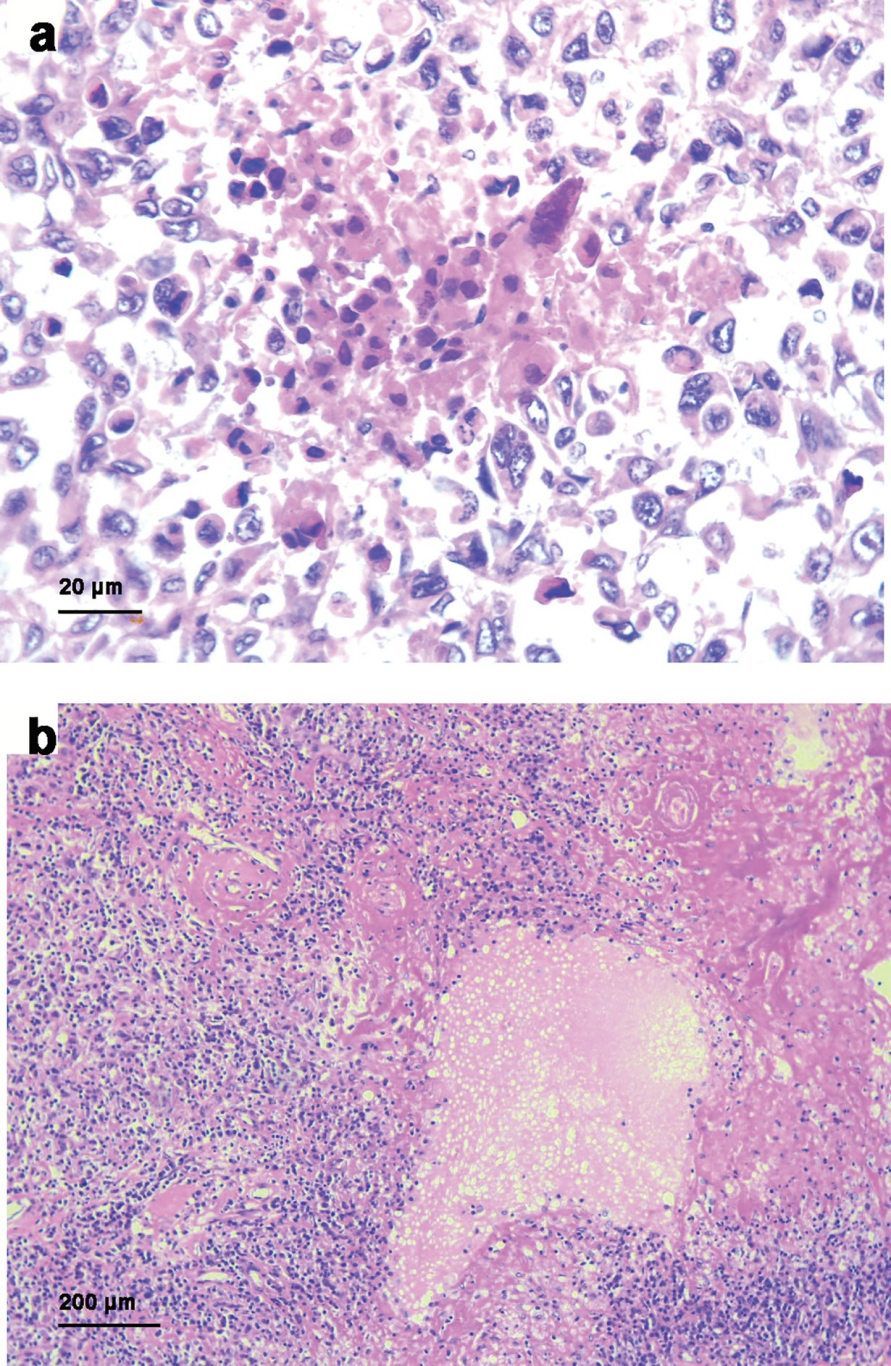
Tumor necrosis. Presence of tumor necrosis in HE-slides of a loco-regional skin metastasis (a) (x 400) and a loco-regional lymph node metastasis (b) (x 40).

Data on tumor necrosis was available in both the primary tumor and the corresponding metastasis in 73 cases. Of these 73 matched cases, 25 cases consisted of primary tumor and corresponding skin metastasis, while the remaining 48 cases consisted of primary tumor and corresponding lymph node metastasis. Among matched cases, tumor necrosis was present in 23 (23 out of 73, 32%) of the primary tumors, compared to 41 (41 out of 73, 56%) of the metastatic tumors (p < 0.01, McNemar test) (S7 Table) (Fig 3).

In skin metastases, the median tumor diameter was 8 mm (range 1–30), and necrosis was present in 8 cases (32%). In lymph node metastases, median tumor diameter was 30 mm (range 1–80) and necrosis was present in 33 cases (69%). Regarding tumor size, necrosis was associated with tumor diameter in skin metastases (p = 0.002), and tended to show a similar association in lymph node metastases (p = 0.055) (Table 4).

**Table 4:** Tumor necrosis in association with VPI and tumor diameter in loco-regional skin and lymph node metastases.

Necrosis in loco-regional metastases (n = 73)			
* *	Absent	Present	p-value^a^
VPI (%) median	6.6	9.2	0.24
**Necrosis in loco-regional skin metastases (n = 25)**			
* *	**Absent**	**Present**	**p-value**^**a**^
Maximum tumor diameter (mm) median	6.5	16.5	0.002
VPI (%) median	11.1	11.7	Ns
**Necrosis in loco-regional lymph node metastases (n = 48)**			
* *	**Absent**	**Present**	**p-value**^**a**^
Maximum tumor diameter (mm) median	23.0	35.0	0.055
VPI (%) median	3.1	8.2	0.015

^a^Mann-Whitney U test

Presence of necrosis was significantly associated with higher VPI in lymph node metastases (p = 0.015), but not in skin metastases (Table 4). There were no significant associations between tumor diameter and VPI in skin- or lymph node metastases (S8 Table).

### Analysis of patient survival

**Fig 4:**
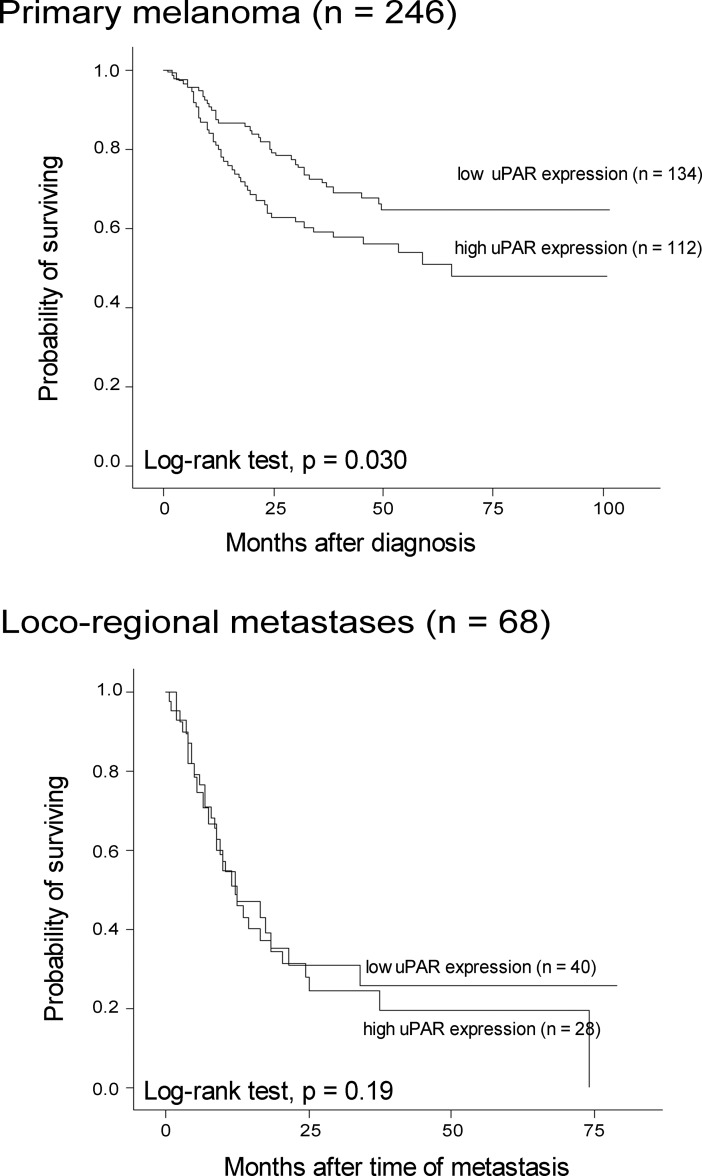
Survival by uPAR expression. Survival by uPAR expression in primary melanoma (upper panel) and metastatic tumors (lower panel), categorized according to the median in primary melanoma (SI 0–3 versus 4–9).

Positive uPAR expression in primary tumors was associated with reduced melanoma-specific survival in univariate analysis (p = 0.03; log-rank test), and showed a similar tendency in metastatic melanoma (p = 0.19, log-rank test) (Fig 4). There was no independent prognostic value of uPAR expression in multivariate analysis (S9 Table).

**Fig 5:**
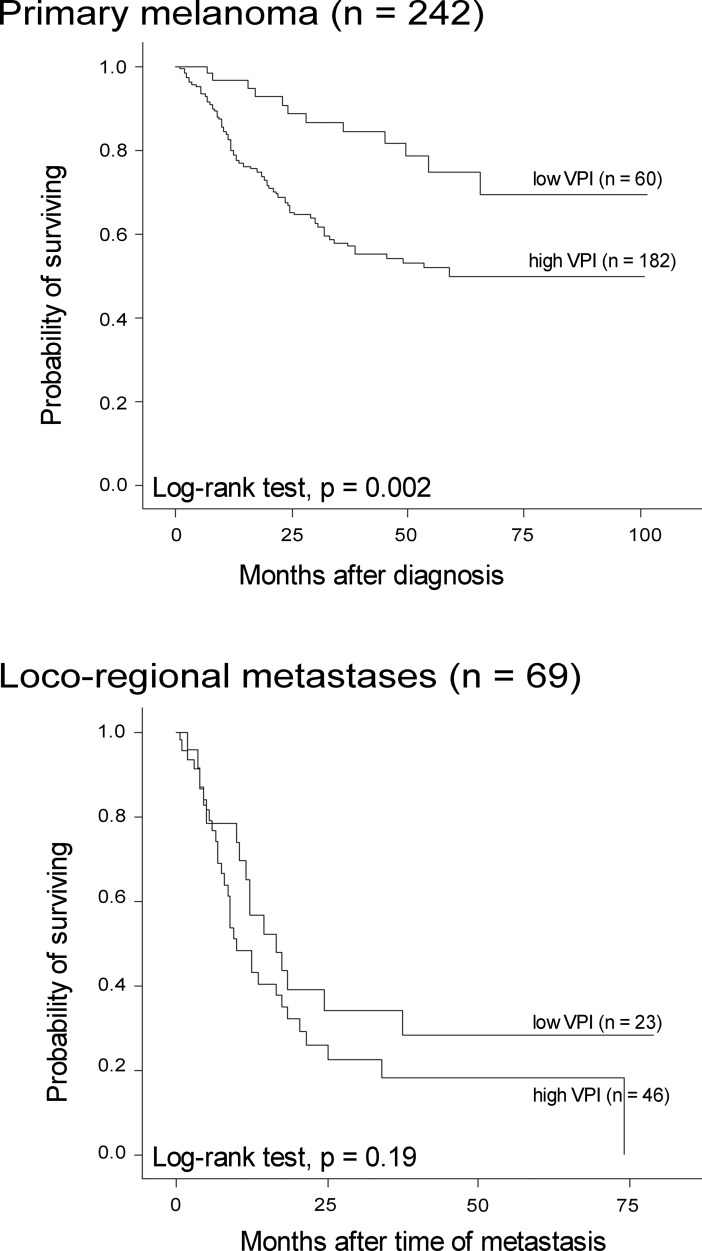
Survival by vascular proliferation. Survival by VPI in primary melanoma (upper panel) and metastatic tumors (lower panel), categorized according to the lower quartile in primary melanoma (5.7%).

VPI in primary melanoma was associated with reduced survival in univariate analysis (p = 0.002; log-rank test), but was not an independent prognostic factor in multivariate analysis when included together with tumor thickness, ulceration, mitotic count and tumor necrosis (S10 Table). There was a similar tendency in univariate analysis of metastatic melanoma, although not significant (p = 0.19, log-rank test) (Fig 5).

**Fig 6:**
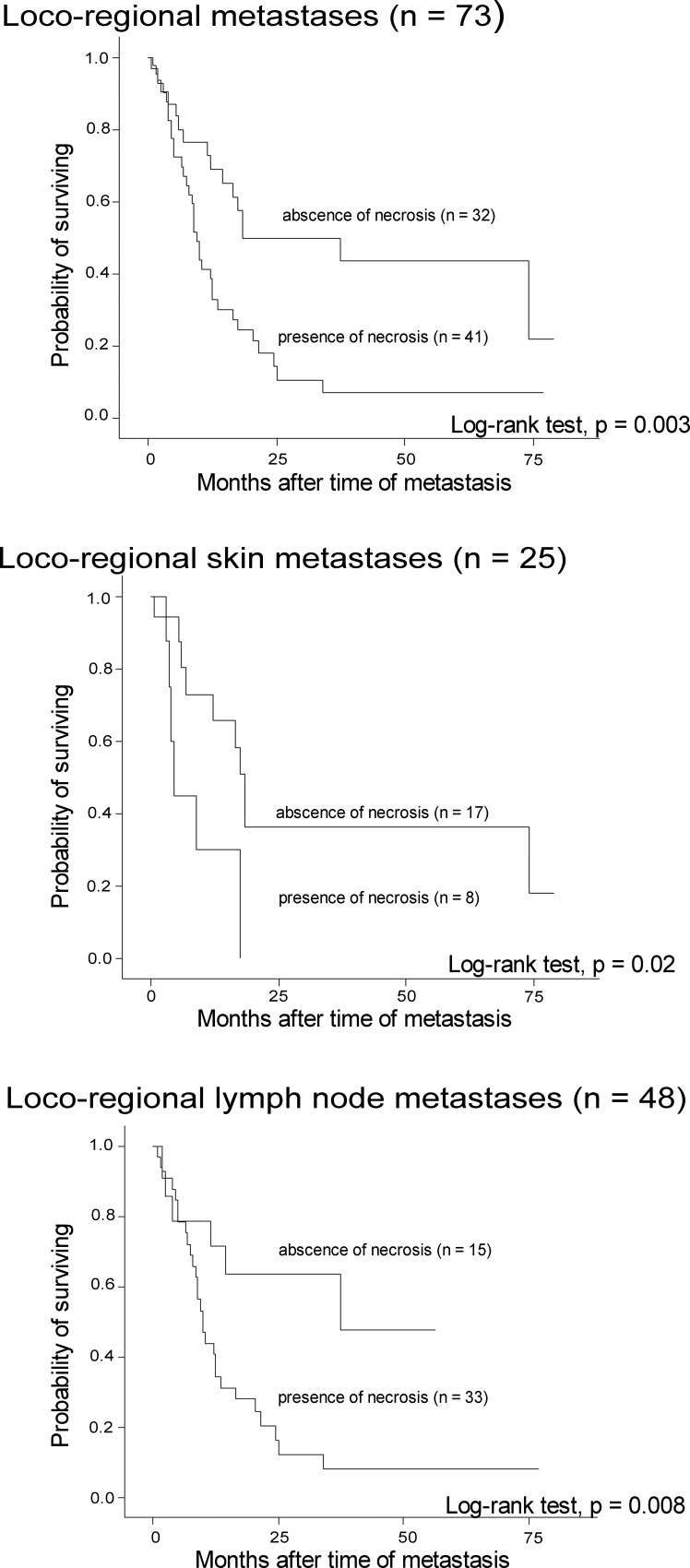
Survival by tumor necrosis. Survival by presence of tumor necrosis in loco-regional melanoma metastases.

Presence of necrosis in melanoma metastases was significantly associated with reduced survival, both when analyzed in all loco-regional metastases (n = 73) (p = 0.003), when analyzed in skin metastases (n = 25) (p = 0.02) and when analyzed in lymph node metastases (n = 48) (p = 0.008) (Fig 6).

Maximum tumor diameter showed a borderline prognostic value in skin metastases (p = 0.06) (lymph node metastases, p = 0.26) (S1 Fig).

## Discussion

We analyzed for the first time a relationship between the vascular proliferation index (VPI), a novel tissue-based angiogenesis marker, and melanoma-specific patient survival, using a large cohort of primary tumors and matched loco-regional metastases. Here, we found a significant impact of VPI on melanoma survival, suggesting that neo-angiogenesis is an aggressive feature of the melanoma microenvironment. This is supported by consistent associations between VPI and high-grade characteristics of the primary tumor: increased tumor thickness, tumor ulceration, higher mitotic count and presence of tumor necrosis. Our finding is in line with a previous report of proliferating endothelial cells associated with reduced overall survival in a small series of primary melanoma [13].

In loco-regional metastases, VPI was lower than in paired primary tumors, with no prognostic impact. These findings indicate that whereas activated angiogenesis appears to be important for expansion of the primary tumor, it might not be as significant at the metastatic stage. In contrast, we found that the MVD level was significantly higher in metastatic lesions than in paired primary tumors. At least for lymph node metastases, a possible explanation for this opposite relationship could be that neo-angiogenesis is not required in the already highly vascularized lymph nodes, and that metastatic tumor tissue might be supported by other mechanisms such as vascular co-option [43]. Interestingly, a previous study from our group demonstrated that VEGF-A was associated with pMVD and VPI in primary melanoma, and with MVD in melanoma metastases [44]. A similar finding of reduced VPI in lymph node metastases compared to primary tumors has recently been shown in breast cancer by our group (Aziz et al, personal communication). In contrast, Pastushenko et al found that the degree of blood vessel proliferation in sentinel lymph node metastases was significantly higher than in primary melanoma, and associated with reduced overall survival [14]. This might indicate a larger demand for neo-angiogenesis in the newly established sentinel lymph node metastases compared to the larger and more advanced non-sentinel lymph node tumors analyzed in our series.

Here, we show for the first time that high expression of uPAR in primary melanoma was associated with reduced patient survival, and related to aggressive tumor features such as increased thickness, mitotic count and tumor necrosis. In addition, uPAR was significantly associated with high VPI, which is consistent with previous reports on the role of uPAR in angiogenesis [22–24]. The functional study by Matheis *et al*. demonstrated that silencing of uPAR lead to apoptosis in melanoma cells, which could support our finding of a prognostic value of uPAR in primary tumors [45]. In loco-regional metastases, uPAR showed no prognostic value or association with VPI, although we found a significant association with overall microvessel density (MVD), indicating that uPAR-driven neo-angiogenesis and invasiveness are more important in primary melanoma than in the loco-regional metastases. In summary, our findings support a role of uPAR in primary melanoma angiogenesis and progression.

Presence of tumor necrosis, an indicator of tumor hypoxia, has shown a consistent association with reduced patient survival in several different cancer types [46], and our group previously reported a prognostic value in primary melanoma, as a novel observation [32, 33]. The present study is the first to investigate tumor necrosis in melanoma loco-regional metastases. We found a striking prognostic value in both skin and lymph node metastases. Presence of necrosis was associated with high VPI in both lymph node lesions and in the primary tumor. Tumor necrosis accompanied by presence of high vascularization has also been observed previously in different cancer types [12, 47–50]. Importantly, tumor necrosis is regarded to develop as a response to intra-tumoral hypoxia [51]. Angiogenesis can be induced by hypoxia directly through HIF1α [52–54], but has also been described as a secondary process to tumor necrosis, induced by inflammatory cytokines from necrotic cells [55–57]. Presence of necrosis was significantly associated with increased tumor diameter in skin metastases and tended to show a similar association in lymph node metastases. This may suggest that inadequate vascularization due to rapid tumor growth is related to the development of tumor necrosis in those tumors. Thus, the adverse prognostic value of tumor necrosis could be due to its relation to fast growing, inadequately vascularized tumors, or to the active role of tumor necrosis in promoting angiogenesis and tumor progression by the release of pro-inflammatory cytokines from necrotic cells [55–57].

In conclusion, we identified high uPAR expression and VPI as novel markers of reduced cancer specific survival in primary melanoma, and confirmed an association of uPAR with angiogenesis. This supports the importance of vascular proliferation in melanoma aggressiveness and progression, and suggests uPAR as an indicator of angiogenesis at least at the stage of primary tumors.

## Supporting information

S1 Fig Survival by maximum tumor diameter in loco-regional metastases, categorized by the higher quartile. (TIF)

S1 Table Patient characteristics (n = 255). (DOCX)

S2 Table Inter-observer agreement for MVD, pMVD and VPI in 25 primary melanoma cases. (DOCX)

S3 Table uPAR expression in association with microvessel density (MVD) and vascular proliferation index (VPI) in loco-regional metastases (n = 68). (DOCX)

S4 Table uPAR^a^ expression in paired primary tumors and loco-regional metastases (n = 65). (DOCX)

S5 Table Distribution of angiogenesis markers (MVD, pMVD and VPI) in primary melanoma according to the presence or absence of loco-regional metastasis (n = 242). (DOCX)

S6 Table Angiogenesis markers (MVD, pMVD and VPI) in paired primary and metastatic melanoma. (DOCX)

S7 Table Tumor necrosis in paired primary tumors and loco-regional metastases (n = 73). (DOCX)

S8 Table Maximum tumor diameter in association with VPI in loco-regional skin and lymph node metastases. (DOCX)

S9 Table Multivariate survival analysis (Cox’ proportional hazards method), with the final model after inclusion of tumor thickness, ulceration, mitotic count and uPAR expression (n = 242). (DOCX)

S10 Table Multivariate survival analysis (Cox’ proportional hazards method), with the final model after inclusion of tumor thickness, ulceration, mitotic count and VPI (n = 239). (DOCX)
