# Supplementary figures and images for: Prognostic value of uPAR expression and angiogenesis in primary and metastatic melanoma
Source: PLoS One. 2019 Jan 14;14(1):e0210399. doi: 10.1371/journal.pone.0210399 (PMC6331131; doi:10.1371/journal.pone.0210399)

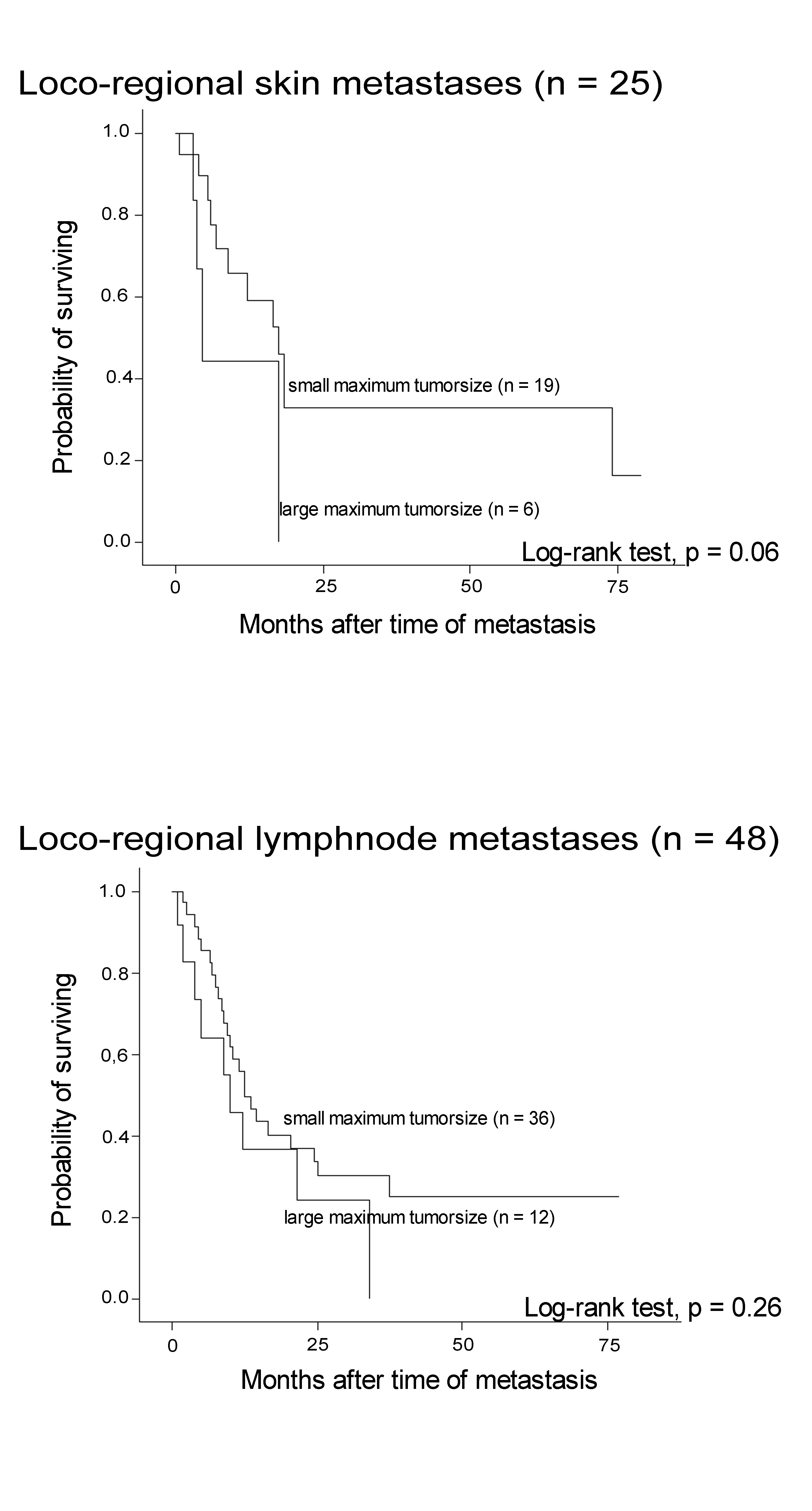

Supplement: S1 Fig — (TIF) [file pone.0210399.s001.tif]
