# Supplementary material for: Prognostic value of uPAR expression and angiogenesis in primary and metastatic melanoma
Source: PLoS One. 2019 Jan 14;14(1):e0210399. doi: 10.1371/journal.pone.0210399 (PMC6331131; doi:10.1371/journal.pone.0210399)
Supplement: S1 Table — (DOCX) [file pone.0210399.s002.docx]

**S1 Table. Patient characteristics (n = 255).**

| Age (years)  Median (range) | 70 (21-98) |
| --- | --- |
| Sex  Men  Women | 138 (54 %)  117 (46 %) |
| Tumor anatomic site^a^  Trunc  Other sites | 77 (31 %)  175 (69 %) |
| Status at last follow-up  Alive  Death from melanoma  Death from other causes | 91 (36 %)  88 (34 %)  76 (30 %) |
