# Supplementary material for: Prognostic value of uPAR expression and angiogenesis in primary and metastatic melanoma
Source: PLoS One. 2019 Jan 14;14(1):e0210399. doi: 10.1371/journal.pone.0210399 (PMC6331131; doi:10.1371/journal.pone.0210399)
Supplement: S2 Table — (DOCX) [file pone.0210399.s003.docx]

**S2 Table. Inter-observer agreement for MVD, pMVD and VPI in 25 primary melanoma cases.**

|  | | |
| --- | --- | --- |
|  | **Registration by IMB** | **Registration by EH** |
| **MVD**  median (no/mm^2^)  kappa | 53.0 | 62.0  0.68 |
| **pMVD** |  |  |
| median (no/mm^2^) | 5.9 | 6.1 |
| kappa |  | 0.61 |
| **VPI**  median (%) | 10.5 | 9.6 |
| kappa |  | 0.84 |
