# Supplementary material for: Prognostic value of uPAR expression and angiogenesis in primary and metastatic melanoma
Source: PLoS One. 2019 Jan 14;14(1):e0210399. doi: 10.1371/journal.pone.0210399 (PMC6331131; doi:10.1371/journal.pone.0210399)
Supplement: S3 Table — (DOCX) [file pone.0210399.s004.docx]

**S3 Table. uPAR expression in association with microvessel density (MVD) and vascular proliferation index (VPI) in loco-regional metastases (n = 68).**

| **uPAR^a^** | | | |
| --- | --- | --- | --- |
|  | **Low** | **High** | **p-value^b^** |
| MVD  median | 91.3 | 126.5 | 0.045 |
| VPI  median | 10.6 | 7.0 | 0.10 |

^a^Categorized according to median staining index in primary melanoma (SI 4)

^b^Mann- Whitney U test
