# Supplementary material for: Prognostic value of uPAR expression and angiogenesis in primary and metastatic melanoma
Source: PLoS One. 2019 Jan 14;14(1):e0210399. doi: 10.1371/journal.pone.0210399 (PMC6331131; doi:10.1371/journal.pone.0210399)
Supplement: S4 Table — (DOCX) [file pone.0210399.s005.docx]

**S4 Table. uPAR^a^ expression in paired primary tumors and loco-regional metastases (n = 65).**

|  | **Primary tumor** | |  |
| --- | --- | --- | --- |
|  | uPAR negative (n) | uPAR positive (n) | p-value^b^ |
| **Loco-regional metastasis** |  |  | ns |
| uPAR negative (n) | 20 (31 %) | 18 (28 %) |  |
| uPAR positive (n) | 11 (17 %) | 16 (25 %) |  |
|  |  |  |  |

^a^Cut-off point: according to median value in primary tumors (SI 0-3 vs 4-9)

^b^Mc Nemar’s test
