# Supplementary material for: Prognostic value of uPAR expression and angiogenesis in primary and metastatic melanoma
Source: PLoS One. 2019 Jan 14;14(1):e0210399. doi: 10.1371/journal.pone.0210399 (PMC6331131; doi:10.1371/journal.pone.0210399)
Supplement: S5 Table — (DOCX) [file pone.0210399.s006.docx]

**S5 Table. Distribution of angiogenesis markers (MVD, pMVD and VPI) in primary melanoma according to the presence or absence of loco-regional metastasis (n = 242).**

|  | **Without loco-regional metastasis**  **( n = 166)** | **With loco-regional metastasis**  **(n=76)** | **p-value^a^** |
| --- | --- | --- | --- |
| **MVD**  median (no/mm^2^) | 59.3 | 68.5 | ns |
| **pMVD**  median (no/mm^2^) | 5.6 | 7.6 | 0.024 |
| **VPI**  median (%) | 8.9 | 10.9 | ns |

^a^Mann-Whitney U test
