# Supplementary material for: Prognostic value of uPAR expression and angiogenesis in primary and metastatic melanoma
Source: PLoS One. 2019 Jan 14;14(1):e0210399. doi: 10.1371/journal.pone.0210399 (PMC6331131; doi:10.1371/journal.pone.0210399)
Supplement: S6 Table — (DOCX) [file pone.0210399.s007.docx]

**S6 Table. Angiogenesis markers (MVD, pMVD and VPI) in paired primary and metastatic melanoma.**

|  | | | |
| --- | --- | --- | --- |
| **n = 67** | **Primary melanoma** | **Loco-regional metastases** | **p-value^a^** |
| **MVD**  median (no/mm^2^) | 67.0 | 97.0 | < 0.001 |
| **pMVD**  median (no/mm^2^) | 7.6 | 10.0 | 0.36 |
| **VPI**  median (%) | 11.0 | 7.6 | < 0.05 |
|  | | | |
| **n = 23** | **Primary melanoma** | **Skin**  **metastases** | **p-value^a^** |
| **MVD**  median (no/mm^2^) | 67.0 | 94.0 | < 0.001 |
| **pMVD**  median (no/mm^2^) | 7.2 | 8.7 | 0.07 |
| **VPI**  median (%) | 11.0 | 10.3 | 0.93 |
|  | | | |
| **n = 44** | **Primary melanoma** | **Lymph node metastases** | **p-value^a^** |
| **MVD**  median (no/mm^2^) | 67.4 | 98.7 | < 0.001 |
| **pMVD**  median (no/mm^2^) | 8.3 | 10.0 | 0.95 |
| **VPI**  median (%) | 11.3 | 7.4 | < 0.05 |

^a^Wilcoxon test
