# Supplementary material for: Prognostic value of uPAR expression and angiogenesis in primary and metastatic melanoma
Source: PLoS One. 2019 Jan 14;14(1):e0210399. doi: 10.1371/journal.pone.0210399 (PMC6331131; doi:10.1371/journal.pone.0210399)
Supplement: S7 Table — (DOCX) [file pone.0210399.s008.docx]

**S7 Table. Tumor necrosis in paired primary tumors and loco-regional metastases (n = 73).**

|  | **Primary tumor** | |  |
| --- | --- | --- | --- |
|  | Necrosis absent (n) | Necrosis present (n) | p-value^a^ |
| **Loco-regional metastasis** |  |  | < 0.01 |
| Necrosis absent (n) | 25 (34 %) | 7 (10 %) |  |
| Necrosis present (n) | 25 (34 %) | 16 (22 %) |  |

^a^McNemar’s test
