# Supplementary material for: Prognostic value of uPAR expression and angiogenesis in primary and metastatic melanoma
Source: PLoS One. 2019 Jan 14;14(1):e0210399. doi: 10.1371/journal.pone.0210399 (PMC6331131; doi:10.1371/journal.pone.0210399)
Supplement: S8 Table — (DOCX) [file pone.0210399.s009.docx]

**S8 Table. Maximum tumor diameter in association with VPI in loco-regional skin and lymph node metastases.**

| **Maximum tumor diameter in loco-regional skin metastases (mm) (n = 25)^a^** | | | |
| --- | --- | --- | --- |
|  | **Small** | **Large** | **p-value^b^** |
| VPI (%)  median | 9.7 | 11.4 | ns |
| **Maximum tumor diameter in loco-regional lymph node metastases (n = 48)^a^** | | | |
|  | **Small** | **Large** | **p-value^b^** |
| VPI (%)  median | 7.2 | 7.8 | ns |

^a^Categorized according to median value

^b^Mann-Whitney U test
