# Supplementary material for: Prognostic value of uPAR expression and angiogenesis in primary and metastatic melanoma
Source: PLoS One. 2019 Jan 14;14(1):e0210399. doi: 10.1371/journal.pone.0210399 (PMC6331131; doi:10.1371/journal.pone.0210399)
Supplement: S9 Table — (DOCX) [file pone.0210399.s010.docx]

**S9 Table. Multivariate survival analysis (Cox’ proportional hazards method). with the final model after inclusion of tumor thickness. ulceration. mitotic count and uPAR expression (n = 242).**

| **Variable** | **N** | **HR** | **95% CI** | **P-value^a^** |
| --- | --- | --- | --- | --- |
| **Tumor thickness (mm)** |  |  |  | 0.02 |
| ≤ 2.0 | 58 | 1 |  |  |
| 2.1-4.0 | 85 | 2.3 | 1.0 – 5.1 |  |
| > 4.0 | 99 | 2.9 | 1.3-6.7 |  |
| **Ulceration** |  |  |  | 0.02 |
| absent | 113 | 1 |  |  |
| present | 129 | 1.8 | 1.1 – 2.9 |  |
| **Mitotic count (no./mm^2)b^** |  |  |  | 0.02 |
| ≤ 1.9 | 56 | 1 |  |  |
| > 1.9 | 186 | 2.2 | 1.1-4.6 |  |
| **uPAR expression (SI)^c^** |  |  |  | 0.09 |
| negative (0-3) | 132 | 1 |  |  |
| positive (4-9) | 110 | 1.0 | 0.7 – 1.6 |  |

Abbreviations: HR: Hazard ratio. CI: Confidence interval

^a^Likelihood ratio

^b^Categorized according to lower quartile

^c^Categorized according to median
